# Five-Year Retrospective Analysis of Superficial Fungal Infections: Insights from Hospital Experience

**DOI:** 10.3390/jof11070474

**Published:** 2025-06-22

**Authors:** Nikoleta Đorđevski, Elizabeta Ristanović, Ana Ćirić, Diana Tomić, Biljana Nikolić, Nemanja Rajčević, Dejan Stojković

**Affiliations:** 1 Institute of Microbiology, Medical Military Academy, Crnotravska 17, 11000 Belgrade, Serbia; nikoleta.djordjevski@gmail.com (N.Đ.); elizabeta.ristanovic@vma.mod.gov.rs (E.R.); drdianatomic@gmail.com (D.T.); 2 Institute for Biological Research “Siniša Stanković”—National Institute of the Republic of Serbia, University of Belgrade, Bulevar Despota Stefana 142, 11000 Belgrade, Serbia; rancic@ibiss.bg.ac.rs; 3 Faculty of Biology, University of Belgrade, Studentski Trg 16, 11000 Belgrade, Serbia; biljanan@bio.bg.ac.rs (B.N.); nemanja@bio.bg.ac.rs (N.R.)

**Keywords:** dermatomycetes, fungal infections, gender differences, temporal variation, mycology

## Abstract

This study aimed to assess the incidence and distribution of dermatomycetes in patients at the Medical Military Academy (MMA) with suspected superficial skin infections over a five-year period (October 2017 to October 2022) and to analyze variations in fungal infections based on factors such as gender, body part, and time, particularly influenced by the COVID-19 pandemic. A total of 3993 samples were analyzed. Collected data were statistically analyzed with two tests. A total of 1048 samples were positive for fungal infections. Over the study period, *Trichophyton mentagrophytes* and *Trichophyton rubrum* were the predominant taxa, while *Microsporum canis* and *Candida albicans* were frequently observed. Statistical analysis indicated significant annual variations for *T. mentagrophytes, T. rubrum*, and *M. canis*, with monthly differences for *T. mentagrophytes* in June and August and *M. canis* in October and December. Gender-based analysis showed that *T. rubrum* and *T. mentagrophytes* were more common in males, while *M. canis*, *C. albicans*, *Candida* spp., and *Geotrichum candidum* were more prevalent in females. Analysis by body part revealed that *Trichophyton rubrum* and *Microsporum canis* showed significant differences between surface types. These findings can help improve diagnostic, therapeutic, and preventative strategies.

## 1. Introduction

The skin is the largest organ of the human body, where the unprotected tissue is directly exposed to the influences of the external environmental factors, including pathogens. This fact and selective pressure contribute to the efficient development and evolution of various defense mechanisms for protection against them [1]. Approximately 20–25% of people worldwide suffer from superficial fungal infections [2], and common fungi linked to the skin have significant carriage rates, even in the absence of obvious disease. For instance, almost 90% of healthy adults have *Malassezia* spp. on their skin [3]. In endemic places, rates of asymptomatic dermatophyte carriage range from less than 1% to 49%, according to research [4]. Although dermatomycoses can be found in all parts of the world, they are especially pronounced in tropical areas due to more favorable conditions for their growth and development, such as higher temperatures and increased humidity. Also, the frequency of dermatomycosis is influenced by a number of other factors such as socioeconomic status, geographic location, seasons, age, gender, etc. [5].

Among dermatomycetes, we can distinguish three ecological groups: geophilic, zoophilic, and anthropophilic [6]. Some species of the genus *Candida* naturally colonize healthy human skin, and only a few of these species can become pathogenic [7,8]. The most common species that leads to symptomatic skin infections from the genus *Canida* is *Candida albicans* [9]. *Malassezia* species commensals of the skin are associated with causing skin infections [10]. The genus *Malassezia* includes 15 species, 12 of which can be found on the skin [11].

Pathogenic fungi infecting the skin are usually confined to the skin and corneal formations. These are the most common surface infections and are rarely life-threatening, but they are quite common and very difficult to treat. Onychomycosis, a superficial infection of the nail bed or plate, accounts for one-third of all fungal infections of the skin [12]. Up to 57% of patients experience a relapse or reinfection with onychomycosis [13]. The fungi that most commonly cause this disease belong to the group of dermatomycetes, the genus *Trichophyton*, and in 80–90% of cases, they are the species *Trichophyton rubrum* and *Trichophyton mentagrophytes* [14]. Onychomycoses are quite common in westernized countries, and they are most often caused by dermatomycetes with a frequency of 80–90%. Yeasts are the cause of this disease with 5–17% frequency, and molds with only 2–3% frequency.

The aim of the present study was to investigate the frequency of superficial skin fungal infections of the patients with supposed symptoms of dermatomycosis, detected and identified at the Institute for microbiology of the Medical Military Academy (MMA), Belgrade, Serbia. The data were collected, presented, and statistically evaluated for a five-year period, from October 2017 to October 2022. The collected data were statistically analyzed using the Mann–Whitney and the Kruskal–Wallis tests. Additionally, diversity indices, including dominance, Simpson’s, and Shannon’s indices, were calculated to assess species diversity across the different samples.

## 2. Materials and Methods

### 2.1. Study Design

This retrospective investigation was carried out at the Military Medical Academy’s (MMA) Institute of Microbiology in Belgrade, Serbia. In all, 3993 clinical samples from patients with suspected superficial fungal infections were gathered over a five-year period from October 2017 to October 2022. Dermatologists sent all of the patients for diagnostic testing. Depending on the infection site, samples were taken from the skin, nails, and hair in the afflicted areas by either skin scraping or nail/hair collection. Relevant metadata were documented, such as sample type, anatomical sampling place, and patient gender. The MMA Ethical Committee accepted the study protocol (Approval No. 4/2021), and all participants provided written informed consent in accordance with the Declaration of Helsinki.

#### Inclusion and Exclusion Criteria

Inclusion Criteria: Male and female patients in a range of age groups (18 years and older) were included in the study; patients with suspected dermatological infections requiring diagnostic testing; and patients from whom samples were taken from skin, nails, or hair in afflicted areas.

Exclusion Criteria: Patients younger than 18 years; patients who have received prior treatment for the dermatological infection that could interfere with diagnostic results; patients with conditions unrelated to dermatological infections that may affect the sample (e.g., systemic diseases affecting skin integrity); and patients who are unable to provide informed consent for participation in the study.

### 2.2. Specimen Sampling

In accordance with the previously mentioned inclusion criteria, samples were taken from individuals who had suspected superficial fungal infections. Depending on the affected area—the skin, nails, or hair—different sampling locations were used.

In order to obtain samples for skin infections, a representative section of the afflicted skin was scraped off using sterile tools. In order to sample both the visible surface and deeper layers of the nail, nail clippings were collected in order to look for nail infections. Hair shafts were extracted from the affected regions of hair in cases of infection, taking care to guarantee that the samples included both the affected and unaffected hair. Every sample was put in a sterile container and labeled with pertinent patient data, such as gender, anatomical location, and sample type. Every sample was sent right away to the lab for additional microbiological examination in the proper conditions.

### 2.3. Species Identification

Pure cultures were isolated and identified by standard microbiological techniques at the Institute for Biological Research “Siniša Stanković”—National Institute of Republic of Serbia, University of Belgrade. The isolates were identified based on colony morphology on Sabouraud dextrose agar (SDA, Torlak, Belgrade, Serbia) and microscopically using morphometric analyses, referred to as “non-MALDI-TOF” identification. The isolates were grown at 25 °C for 72 h in an incubator. Growth media (SDA), colony and mycelium characteristics (size, shape, texture, and color), and microscopic features (spore morphology, spore size, and shape) are used to identify fungi morphologically. When paired with other data (based on the clinical history and infection site), those criteria enable a successful morphological identification of fungal species. Nonetheless, MALDI-TOF confirmation is frequently required for accurate species identification.

The basis of the MALDI-TOF MS approach is the detection of fungal protein mass spectra peculiar to a species. After being incubated for 24 to 72 h, a pure colony of fresh fungal isolate was spread out on the VITEK^®^ MS-DS target slide spot. On the dried smear, 1 µL of matrix solution (CHCA matrix, bi-437 oMerieux SA, Marcy-I’Etoile, France) was applied. The prepared samples were exposed to laser beams, ionized and evaporated, and then passed through a vacuum tube’s electric field to a linear detector, which produced a mass spectrum. A good confidence value (60 to 99.9% likelihood) was achieved by comparing this spectrum with the spectrum in the knowledge base (VITEK^®^ MS V3.3 knowledge base-bioMérieux), which allowed for the identification of fungal species in the investigated sample. The process was repeated for isolates with poor discrimination or no identification findings, but an extra step was added: 70% formic acid was added prior to matrix solution for a more effective protein extraction. No additional testing was performed on isolates with a second unsuccessful identification result. To keep with clinical and standard terminology, we refer to the species as *Candida guilliermondii* throughout the text, even though its current classification is *Meyerozyma guilliermondii* (Wick.) Kurtzman & M. Suzuki.

### 2.4. Statistical Analysis

Collected data were statistically analyzed with two tests: Mann–Whitney (M–W) (pairwise comparison) and Kruskal–Wallis (K–W) (comparison for more than two samples). Univariate descriptive statistics were used to study the raw data. A presence/absence matrix of the taxa was used, where presence was coded as 1 and absence as 0. Non-parametric tests were used to test for differences between a priori groups, namely Kruskal–Wallis (comparison for more than two samples) and Mann–Whitney for comparison of two samples and as a post hoc pairwise test in multiple group comparison, with *p* < 0.05 as a cut-off for statistical significance. Results of the K–W and M–W tests were confirmed using ε^2^ and power analysis [15]. Diversity indices were also calculated (dominance, Simpson’s and Shenon’s diversity indices) to account for the species diversity of different samples. Approximate confidence intervals for all these indices were computed with a bootstrap procedure. For each individual in the random sample, the taxon was chosen with probabilities proportional to the original abundances to calculate a 95 percent confidence interval. All statistical analyses were performed using PAST 4.17 [16].

## 3. Results

This study monitored the incidence of skin pathogens including dermatomycetes, yeasts, and other rare filamentous fungal pathogens in patients visiting the MMA over a five-year period from October 2017 to October 2022. Out of a total of 3993 patients referred for microbiological testing due to clinical suspicion of fungal infection, only 1048 (26.2%) had laboratory-confirmed fungal etiology. The remaining samples (73.8%) tested negative for fungal presence, which can be attributed to the fact that some patients exhibited skin changes that clinically resembled fungal infections but were actually due to other dermatological conditions, such as dermatitis or bacterial infections. In this study, we analyzed only those samples that tested positive for fungal pathogens. Raw data for identified fungi from the year 2017–2018 are presented in the Supplementary Material. These data includes information on patient gender as well as the affected body part and surface. Data were collected for each month of the year, detailing the total number of samples, including both positive and negative outcomes.

### 3.1. The Ratio of Taxa in Positive Samples per Year, Irrespective of Gender

**Figure 1:**
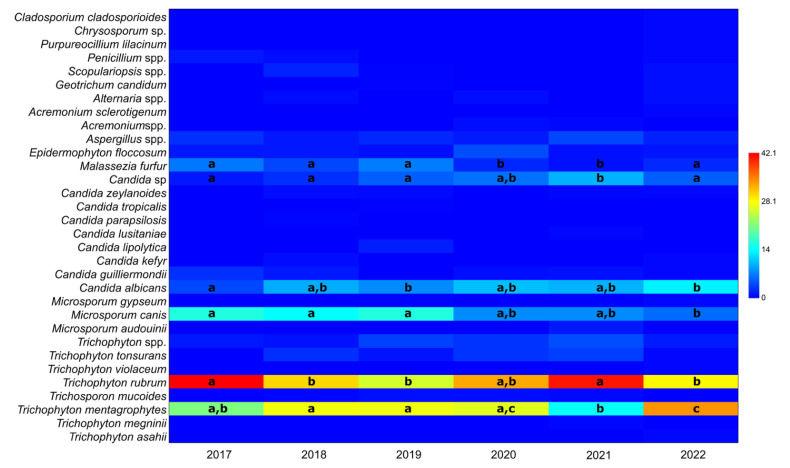
The ratio of taxa in positive samples per year, irrespective of gender. Frequencies with same letters within the same row do not differ significantly (Mann–Whitney pairwise tests). No letters were assigned for rows that did not show statistically significant differences in the Kruskal–Wallis test.

Due to the substantial variation in the total number of samples per year caused by COVID-19 (2020–2021 compared with other years), the number of samples was calculated as a percentage of the total positive samples for each year. The relative distribution of taxa in positive samples per year, irrespective of gender, is presented in Figure 1.

Throughout the study period, two taxa consistently dominated: *T. mentagrophytes* and *T. rubrum*, which comprised 25.1 ± 6.6% and 33.4 ± 6.7% of the positive samples, respectively. Two other taxa were also frequently observed: *Microsporum canis* (11.2 ± 4.6%) and *C. albicans* (9.1 ± 3.2%). The frequency of these two taxa varied over the years, with the frequency of *C. albicans* increasing and that of *M. canis* decreasing (Table 1).

The nonparametric K–W test indicated statistically significant differences in the occurrence of *T. mentagrophytes* and *T. rubrum* across different years (χ^2^ = 16.31, *p* = 3.68 × 10^−5^ and χ^2^ = 14.16, *p* = 5.65 × 10^−4^, respectively). The significance of the K–W test was confirmed by ε^2^, where values for every tested taxon ranged from 0.0015 to 0.012, indicating very small to medium effect size [15]. The M–W pairwise tests revealed that statistically significant differences were found only between specific years: for *T. mentagrophytes*, between 2021 and 2022, and for *T. rubrum*, between 2017 and 2021. It is important to note that data from 2017 and 2022 are partial, as the study period was from October 2017 to October 2022.

Similar results were observed for *M. canis*, with significant differences between the years 2017–2019 and 2020–2022. No statistically significant differences were found for the remaining taxa, except for *Candida* spp. and *Malassezia furfur*, where 2021 differed significantly from all other years.

### 3.2. Number of Taxa in Positive Samples per Month, Irrespective of Year or Gender

**Figure 2:**
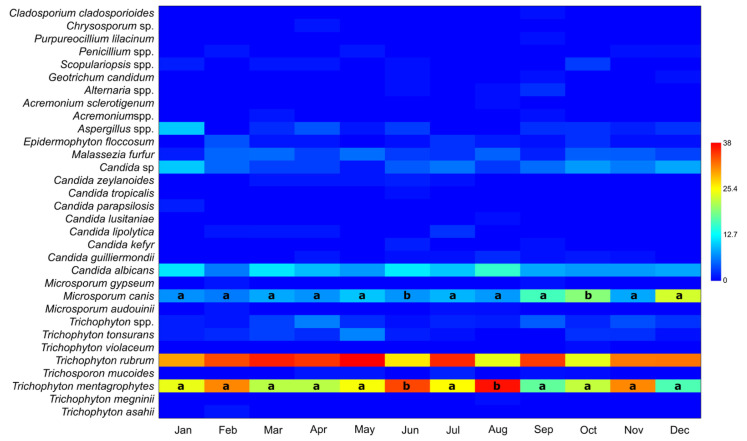
Number of taxa in positive samples per month, irrespective of year or gender. There were no statistically significant differences except in only a few cases, as indicated by the same letter.

The number of taxa in positive samples per month, irrespective of year or gender, is presented in Table 2 and Figure 2. Although the same six taxa appear across different months, their monthly distributions vary. The Kruskal–Wallis (ε^2^ were small, 0.010 and 0.013 for *M. canis* and *T. mentagrophytes*, respectively) and post hoc Mann–Whitney tests did not reveal statistically significant differences in the distribution of taxa between months, except for *T. mentagrophytes* in June and August and *M. canis* in October and December. These months were statistically different from all the others.

Most taxa were rare, with 22 taxa having 10 or fewer positive samples out of a total of 1409. Therefore, significant differences between genders were not commonly observed. However, a few taxa did show statistically significant differences in abundance between genders: *T. rubrum* and *T. mentagrophytes* were more abundant in males, while *M. canis*, *Candida* spp., and *Geotrichum candidum* were more abundant in females.

### 3.3. Gender-Based Abundances of Taxa

**Figure 3:**
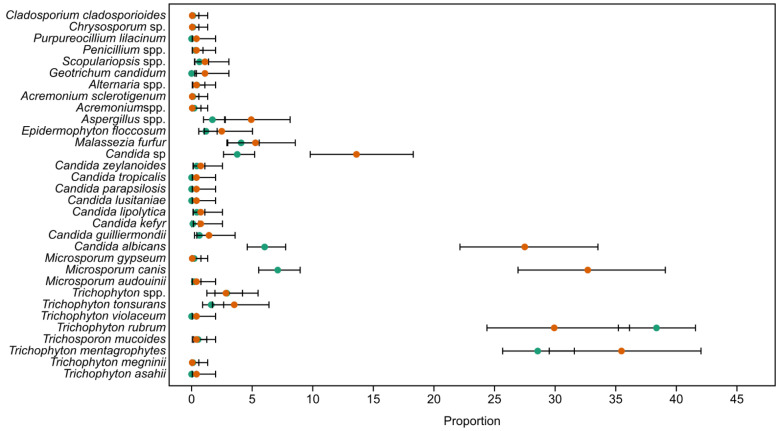
Gender-based frequencies of taxa. Whiskers represent the 95% confidence interval. Statistically significant differences based on the results of Kruskal–Wallis test. Orange dots female, green dots males.

Gender-based frequencies of taxa are shown in Figure 3. *p*-values from the Kruskal–Wallis test, when less than 0.05, are also included in Figure 3. The strength of the analysis was confirmed using an ε^2^ test (0.0013–0.028) that showed a very small to small effect size on the results of the K–W test, while the power analysis showed between 86.4 and 100% power for α = 0.01, indicating the significance of the obtained results. Due to large bias toward men in this analysis, frequencies were used instead of raw data. Because there was a large number of taxa that had only few occurrences, power analysis was also performed on all taxa. Sample size was good for all of the taxa that had statistically significant differences (86.4–100.0%, α = 0.05), but for most of the rare taxa, power analysis was low, ranging from 2.6% for *Candida zeylanoides* and *C. lipolytica* to 33.9% for *Trichophyton asahii, Candida lusitaniae, C. parapsilosis*, and *C. tropicalis*.

### 3.4. Ratio of Species by Body Part

**Figure 4:**
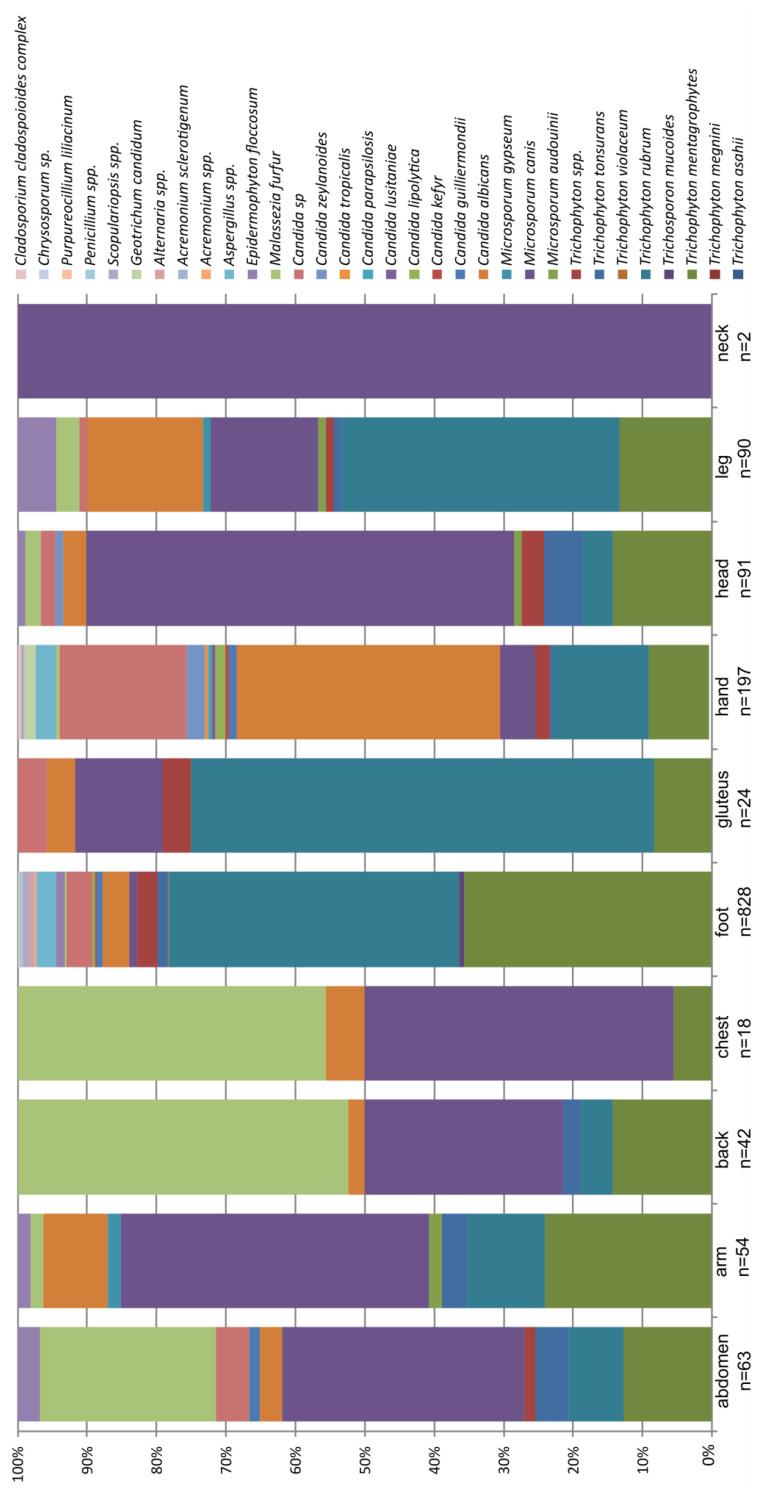
Ratio of species by body part (%). n—sample size.

The ratio of species by body part (%) is presented in Figure 4 and Table 3. Foot and neck were the most distinctive among all the sampling areas, neck due to a very low number of samples and foot due to it being the largest number of positive samples. *T. rubrum* dominated the samples obtained from foot, gluteus, and leg, while *M. canis* dominated samples from abdomen, chest, neck, and head. *Malassezia furfur* was the most abundant in the samples from back and chest, while *C. albicans* was the most common in samples from hands.

The Kruskal–Wallis test showed statistically significant differentiation of body parts for eight taxa. However, a pairwise Mann–Whitney test did not show separation of all body parts for each of these taxa, but rather a grouping of body parts, or, separation of one body part from all the others.

Difference in the domination of certain taxa (e.g., *Trichophyton, Malassezia*, and *Candida*) was confirmed with these tests.

### 3.5. Ratio of Taxa per Surface Type

**Figure 5:**
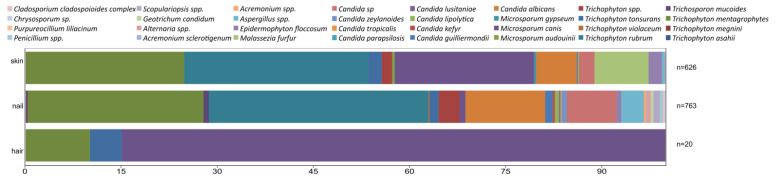
Ratio of taxa per surface type (%). n—sample size.

There were some differences in the presence of taxa per surface type. The biggest difference can be seen on hair, but this is expected due to the low number of these samples. Nail and skin showed a somewhat similar ratio of different taxa, though several groups were present only in the nail and not the skin, which cannot be entirely explained regarding the number of samples, as the numbers were not that much smaller for skin. Table 4 and Figure 5 present results for the ratio of taxa per surface type (%).

The Kruskal–Wallis test showed statistically significant differences between surface types only in six taxa. However, the post hoc pairwise Mann–Whitney test was able to significantly separate all of the surfaces for only *T. rubrum* and *M. canis*, while in all the other taxa, only skin and nail were significantly different.

### 3.6. “Non-MALDI-TOF” and MALDI-TOF-Detected Samples

**Figure 6:**
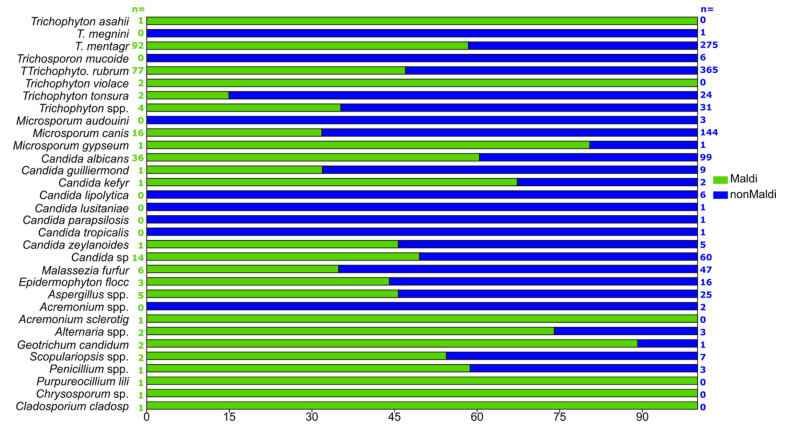
“Non-MALDI-TOF” and MALDI-TOF-detected samples. n—number of samples of each taxon for “non-MALDI-TOF” and MALDI-TOF.

Differences between “non-MALDI-TOF“ and MALDI-TOF-detected samples are presented in Figure 6. Because the MALDI was used only in 2022, frequencies of detected taxa were calculated within each group. Based on the frequencies of detection, there are some differences between these two methods of identification.

For example, *Trichophyton megnini*, *Trichosporon mucoides*, *Microsporum audouinii*, *Candida lipolytica*, *Candida lusitaniae*, *Candida parapsilosis*, *Candida tropicalis*, and *Acremonium* spp. were not detected when using MALDI-TOF. This is most probably due to the fact that most of the samples were extremely rare in the total sample (had only one or two instances), with only *T. mucoides* and *C. lipolytica* that were present in a total of six samples.

On the other hand, *Trichosporon asahii*, *T. violaceum*, *Acremonium sclerotigenum*, *Purpureocillium liliacinum*, *Chrysosporum* sp., and *Cladosporium cladospoioides* complexes were detected only by using MALDI-TOF, though all of these were detected only once with a frequency of 1 in every 270 samples. For all other taxa, approximately the same ratios were detected or, in all but one case, there were slightly higher numbers of occurrences for MALDI-TOF detection.

### 3.7. Diversity Index

Twenty-six different taxa were present in samples collected from females, in contrast to 24 found in males, even though two-thirds of the samples was coming from the male patients. This means that there is a higher diversity of taxa present in samples from female patients, as indicated by Shannon’s diversity, coupled with lower dominance of a single taxon. Shannon’s diversity index usually ranges from 1.5 to 3.5, where values below 1.5 would indicate low diversity. The Simpson’s index also describes diversity. In the present study, most of the samples had a Simpson’s index higher than 0.61, indicating a moderate to high degree of diversity, with the exception of hair, which had low diversity.

Diversity index results are presented in Table 5 (with respect to colonized body part) and Table 6 (with respect to month of isolation). When looking at body parts, most of the taxa were detected on the feet and the hands, though the highest number of samples came from these body parts. Nevertheless, the highest diversity indices were detected for hands, followed by those for the abdomen and legs, while feet showed only the fifth highest diversity. There was not a single sample that was strongly dominated by a single taxon, though higher grouping type was detected in comparison with gender. Shannon’s and Simpson’s diversity indices suggest high diversity of taxa irrespective of month of sampling.

When analyzing the diversity of surfaces, the lowest diversity was found for hair, while the highest was for nail, which is congruent with the previous analysis. Of the three taxa that were detected in samples taken from hair, there was a stronger grouping type of a single taxon as indicated by the dominance index.

On the other hand, there was no difference in the samples in their diversity based on the months the samples were taken, where most show moderate diversity and a similar number of detected taxa.

## 4. Discussion

Approximately 10% of hospital-acquired infections are caused by micromycetes. Among human and animal pathogens, the group of dermatomycetes is the main cause of dermatomycoses, infections that are chronic, not fatal, but lead to significant morbidity [17]. Statistical studies of the frequency of dermatomycetes are of crucial importance in order to gain insight into the frequency of their occurrence among the population. However, such studies are rarely conducted in our country and region. There are several recent statistical analyses of infections caused by bacteria and yeasts, while analyses of infections caused by dermatomycetes are much rarer [18].

The treatment of superficial mycoses is significantly impacted clinically by the precise identification of particular fungus species. For instance, the most common species in our study, *Trichophyton rubrum* and *T. mentagrophytes*, are known to be linked to recurring and chronic illnesses such onychomycosis and tinea pedis [19]. In certain geographical areas, these species have shown varying susceptibilities to antifungal medications, and they frequently need long-term therapy [20]. We acknowledge that antifungal resistance in the terbinafine-resistant strain of *T. indotineae* is an increasing global problem [21]. There are currently no verified reports of *T. indotineae*’s existence in Serbia, despite the fact that it has caused significant outbreaks in South Asia and has later been found in a number of European nations (such as France, Sweden, and Germany) and North America [21,22]. Because non-*C. albicans* may show less sensitivity to widely used azoles, requiring alternate treatment regimens, it is also therapeutically relevant to identify *Candida albicans* and other *Candida* species [23]. Furthermore, the development of antifungal resistance emphasizes the necessity of species-specific diagnostic methods to direct efficient therapy and lower the likelihood of treatment failure, especially in patients with immunocompromised conditions or repeated cases [23]. Routine species-level identification can minimize empirical treatment, optimize therapeutic choices, and enhance patient outcomes when incorporated into diagnostic workflows [24].

Our study addressed a critical area of concern in infectious disease. The contemporary use of statistics in analyzing superficial fungal infections provides a novel perspective that bridges clinical practice and computational analysis. Additionally, the study’s findings are relevant not only to healthcare professionals but also to researchers and policymakers focused on improving fungal infection management globally. The insights gained from this research can help enhance diagnostic, therapeutic, and preventative measures, making it a significant contribution to the field.

Our results for the ratio of taxa in positive samples per year (Figure 1) differ from those reported in earlier decades. For instance, in the 1960s and 1970s, scalp infections in Eastern Europe were predominantly caused by *T. violaceum* and *T. schoenleinii*, with *M. audouinii* being particularly prevalent in Serbia and Romania. In contrast, our study found *T. rubrum* and *T. mentagrophytes* to be the most common species, aligning more closely with Western European trends from the same historical period [25]. More recently, a study from Niš, Serbia, reported *M. canis* as the dominant species, with a prevalence of 63.9% [18] compared with our finding of 11.2 ± 4.6%. These discrepancies may reflect regional differences in animal contact, environmental exposure, or healthcare practices.

Figure 2 and Table 2 present our results for number of taxa in positive samples per month, irrespective of year or gender. In a study conducted in Germany during 2020/2021 [26] and presenting the taxa distribution across months, the highest frequency of occurrence was *Trichophyton benhamiae* for the months from June to September and *Trichophyton quinckeanum* from September to January. The last one (*T. quickeanum*) is a dermatomycete whose significantly increased occurrence is recorded since 2014/2015. On the contrary, the results of this study together with the previously published data [18] showed it has not been presented in our country at all. Opposite to our study, *T. mentagrophytes* had the highest increase in occurrence in September, while *M. canis* had a negligible increase in November [27]. This can lead us to the conclusion that during the years affected by the COVID-19 pandemic in different countries, the distribution of the most common dermatomycetes was very variable, both in relation to the occurrence during years and in relation to the distribution in the same months during one year. A mix of environmental and behavioral factors may be responsible for the seasonal fluctuations in the prevalence of several fungal species that have been documented, such as the higher occurrence of *T. mentagrophytes* in June and August and *M. canis* in October and December. Fungal growth and transmission are facilitated by warmer temperatures and higher humidity levels in the summer, especially in shared or damp spaces like gyms, locker rooms, and swimming pools [27]. Dermatomycoses may also be more common during these times due to increased outdoor activity and skin surface exposure. On the other hand, the increased usage of occlusive clothes and footwear during the winter months may encourage the growth of fungi in places like the feet. Furthermore, as pets are known to be reservoirs for this zoophilic species, the autumn and winter surge in *M. canis* may be due to increased indoor interaction with them. The significance of combining public health awareness efforts with infection control tactics suited to particular seasons of the year is underscored by these seasonal patterns [24].

Considering our results for gender-based abundances of taxa presented in Figure 3, one interesting observation appeared: the number of samples from males was almost twice the number of samples taken from females, and most of those came from two species of *Trichophyton*. The distribution of dermatomycetes by gender is different in different countries. For example, superficial skin infections (SFIs) caused by dermatomycetes in Korean population, according to the KNHIS (Korean National Health Insurance Service) for the period ranging from 2009 to 2018, occur more often in men compared with women (62.59% vs. 55.56%; *p*  <  0.001) [25]. On the contrary, the frequency of infections caused by these fungi in Slovakia (55.75% vs. 44.25%) and Brazil (61.4% vs. 38.6%) was higher in women [28,29]. A possible reason for male predominance in our study is more frequent hospital visitation by men as we conducted our study in a military hospital, and the majority of people employed in the Serbian army are men [30]. Because of this, the dataset might not accurately depict how superficial fungal infections are distributed by gender in the overall population. As a result, results pertaining to gender prevalence should be read cautiously and seen as indicative of a particular institutional population rather than the general civilian population.

The results for ratio of species by body part (%), which are presented in Figure 4, corresponded with those found in the literature, indicating that the most common infected area was foot [28,31]. The diversity of samples was also the highest in foot. Considering the number of samples, it was also the highest in this area, and for that reason, such a result was expected. The results of our work for *T. rubrum* were consistent with those in the literature, where this fungus was cited as the most common cause of *tinea pedis* [32].

*M. canis* is sometimes transmitted by infected cats and dogs, which are the main two reservoirs of this dermatomycetes in the mentioned order [33]. Considering that this fungus is most commonly transmitted by direct contact with a sick animal or its feces, our findings of the most common occurrence of *M. canis* on the neck, head, chest, and abdomen were to be expected.

As for the yeasts, *Malassezia* is most commonly found on seborrheic skin areas, ears, scalp, and breasts as it lacks the genes to synthesize fatty acids and therefore rely on exogenous lipids. However, this yeast can be found on all other parts of the body except the feet. Therefore, our data were consistent with those from the literature [34]. Another yeast, *C. albicans*, is a fungus most commonly found on the mucous membranes of the mouth, genital area, and gastrointestinal tract, but it can also infect the skin, usually only the uppermost layer of the skin, the *stratum corneum*. However, the second most common skin infection with *C. albicans* occurs precisely on the hands [35].

Even though the Shannon, Simpson, and dominance indices were developed to describe species diversity, they are still mostly used for descriptive purposes today. Correlating diversity with clinical or demographic characteristics, for example, could greatly increase the findings’ epidemiological value. For instance, while having fewer samples, the higher taxonomic diversity seen in female patients may indicate underlying variations in exposure patterns, hygiene habits, or host sensitivity [36]. Similarly, the observed disparities in diversity between body areas may be due to variances in moisture retention, skin microenvironments, or the frequency of environmental interaction. More reliable ecological conclusions could be performed by combining diversity indices with stratified information on age, comorbidities, anatomical sampling locations, or infection recurrence. Additionally, by identifying particular risk groups or body areas that are more likely to be colonized by a wider variety of fungal species, these analyses could help guide tailored treatment plans and diagnostic priorities [37].

The results of our study, presented in Figure 6, were to be expected because the microbiological method, the direct microscopy method, can be a less sensitive analysis in clinical practice, and the MALDI-TOF measurement is a more specific and precise method.

The results of this study should be interpreted with some caveats in mind. First, the sample population was selected from the Military Medical Academy, where the majority of the patients are military people. This could limit the data’s generalizability to the larger civilian population and introduce selection bias. Second, although culture remains the routine diagnostic standard in many clinical laboratories, it is known to have limited sensitivity, especially in cases of prior antifungal use, low fungal burden, or suboptimal sampling. Third, there were methodological differences during the study period, especially because MALDI-TOF identification was not introduced until 2022. This might have caused some taxa to be overrepresented or underrepresented in previous years. Furthermore, certain species might not have been identified because of the MALDI-TOF MS reference database’s shortcomings, which could include incomplete spectral information for uncommon or newly discovered fungal taxa, which could result in underidentification or incorrect categorization, such as species-level resolution within the *Trichophyton mentagrophytes* complex. The VITEK^®^ MS V3.3 knowledge base-bioMérieux software is capable of differentiating between *T. mentagrophytes* and *T. interdigitale*. However, we acknowledge that the distinction from *T. indotineae* may remain uncertain, as this species is not yet reliably distinguished in many commercial MALDI-TOF libraries. Fourth, the retrospective approach made it unable to gather comprehensive clinical information that would have enabled more in-depth associations with species distribution, such as comorbidities, duration, severity, or previous antifungal therapies. Furthermore, the observed temporal and anatomical variations may have been caused by behavioral and environmental factors that were not rigorously evaluated, such as seasonal dress choices, pet ownership, or hygiene practices. The necessity for prospective, multicenter trials with more thorough clinical and environmental data is highlighted by these constraints.

## 5. Conclusions

The study presented a detailed five-year retrospective analysis of superficial fungal infections, utilizing bioinformatics tools to assess trends and patterns. Findings offer valuable insights into the epidemiology of superficial fungal infections, identifying prevalent species, which can inform clinical practices and patient management. The research is grounded in real-world data from a hospital setting, ensuring relevance to clinical practitioners and contributing to a broader understanding of fungal infections in healthcare environments. Given the increasing prevalence of fungal infections worldwide, the findings have implications for global health, particularly in resource-limited settings.

We advise routine use of MALDI-TOF mass spectrometry for accurate, species-level identification of superficial fungal infections due to the observed variability in species distribution and diagnostic accuracy. Furthermore, the integration of molecular diagnostics—such as PCR-based assays or next-generation sequencing—offers even higher sensitivity and the ability to detect non-viable organisms or mixed infections. We recommend incorporating molecular tools to enhance diagnostic accuracy, enable earlier detection, and support surveillance for antifungal resistance genes. Our results suggest that while diagnosing and treating superficial fungal infections, doctors should take into account the patient’s gender, the time of year, and the site of infection. Males and lower limbs were more likely to have *T. rubrum* and *T. mentagrophytes*, whereas females and upper body regions were more likely to have *M. canis* and *Candida* spp. Additionally, seasonal increases point to the necessity of heightened awareness in the summer and fall. To lower the chance of recurrence, patients are encouraged to practice hygiene, refrain from sharing personal belongings, keep an eye out for skin problems in their pets, and take their antifungal medications as directed. Because terbinafine is still the most effective oral medication, the possible introduction of *T. indotineae* or resistant genotypes may have a substantial effect on the effectiveness of treatment. Therefore, in order to identify resistance trends early, we advise integrating antifungal susceptibility tests (such as squalene epoxidase gene sequencing or MIC determination) into future surveillance initiatives.

## Figures and Tables

**Table 1:** Frequencies and confidence interval (CI_95_) of positive occurrences of most abundant taxa per year, irrespective of gender.

Taxon	2017	2018	2019	2020	2021	2022
*Trichophyton mentagrophytes*	21.1 (0.13–0.32)	27.4 (0.23–0.33)	27.2 (0.23–0.32)	26.6 (0.19–0.35)	14.5 (0.10–0.20)	33.9 (0.28–0.40)
*Trichophyton rubrum*	42.1 (0.31–0.54)	30.3 (0.25–0.36)	25.6 (0.21–0.30)	32.9 (0.25–0.42)	40.9 (0.35–0.48)	28.4 (0.23–0.34)
*Microsporum canis*	15.8 (0.08–0.26)	14.2 (0.11–0.19)	15.8 (0.12–0.2)	7.7 (0.04–0.13)	7.7 (0.04–0.12)	5.9 (0.03–0.09)
*Candida albicans*	3.9 (0.01–0.11)	9.5 (0.06–0.13)	7.6 (0.05–0.11)	10.5 (0.06–0.17)	9.8 (0.06–0.14)	13.3 (0.09–0.18)
*Malassezia furfur*	6.6 (0.02–0.15)	3.8 (0.02–0.07)	6.8 (0.04–0.10)	2.1 (0.00–0.06)	0.9 (0.00–0.03)	2.2 (0.01–0.05)
*Candida* sp.	1.3 (0.00–0.07)	2.5 (0.01–0.05)	5.2 (0.03–0.08)	6.3 (0.03–0.12)	9.8 (0.06–0.14)	5.2 (0.03–0.09)

CI_95_ intervals are given in parentheses.

**Table 2:** Number of taxa in positive samples per month, irrespective of year or gender.

Taxon	χ^2^	*p*	
*Trichophyton mentagrophytes*	18.27	0.000978	June/August diff.
*Trichophyton rubrum*	10.23	0.14	No diff.
*Microsporum canis*	13.6	0.00000644	Oct/Dec diff.
*Candida albicans*	2.65	0.53	No diff.
*Candida* sp.	2.28	0.18	No diff.
*Malassezia furfur*	0.72	0.84	No diff.

**Table 3:** Ratio of species by body part (%).

Taxon	X^2^	*p*	ε^2^	Mann–Whitney
*Trichophyton mentagrophytes*	60.12	3.17 × 10^−18^	0.04	More common in foot; foot differed from all other parts, except neck and arm
*Trichophyton rubrum*	108.3	2.71 × 10^−31^	0.08	More common in foot; foot differed from all but leg. Gluteus differed from all other parts by a low frequency.
*Microsporium cannis*	140	1.32 × 10^−92^	0.10	Almost all of the body parts showed statistically significant differences.
*Microsporium gypseum*	0.08	2.98 × 10^−2^	<0.001	Only foot was different from leg and arm as it was present only in these body parts. No other differences.
*Candida albicans*	61.85	2.28 × 10^−45^	0.04	Only hand was different from all other body parts due to high frequency.
*Candida zeylanoides*	0.34	1.6 × 10^−3^	<0.001	Only foot was different from hand and head. No other differences due to absence of detection on other body parts.
*Candida* sp.	12.66	3.53 × 10^−14^	0.010	Only hand was different from all other body parts due to higher frequency.
*Malassezia furfur*	46.22	1.93 × 10^−84^	0.03	Back, abdomen, and chest were different from all other parts and between each other due to varying and higher abundances.

**Table 4:** Ratio of taxa per surface type (%).

Taxon	X^2^	*p*	ε^2^	Mann–Whitney
*Trichophyton rubrum*	9.4	7.1 × 10^−4^	0.007	Complete differentiation of all surfaces.
*Microsporium cannis*	77.6	1.29 × 10^−55^	0.056	Complete differentiation of all surfaces.
*Candida albicans*	4.61	1.63 × 10^−5^	0.003	Skin and nail were different from hair due to absence in hair. More commonly found on nail.
*Candida* sp.	3.25	2.29 × 10^−05^	0.002	Only skin and nail were different due to absence in hair. More commonly found on nail.
*Malassezia furfur*	7.52	1.74 × 10^−15^	0.005	Found only on skin.
*Aspergillus* spp.	1.03	3.07 × 10^−5^	<0.001	Only skin and nail were different, with higher frequency on nail and due to absence in hair.

**Table 5:** Diversity index (with respect to colonized body part).

	Male	Female	Abdomen	Arm	Back	Chest	Foot	Gluteus	Hand	Head	Leg	Neck	Skin	Nail	Hair
Number of taxa	24	26	10	9	6	4	23	6	19	11	11	1	15	28	3
Individuals	927	480	63	54	42	18	828	24	196	91	90	2	626	762	20
Dominance	0.242	0.151	0.203	0.266	0.3159	0.366	0.306	0.449	0.208	0.401	0.226	1	0.203	0.218	0.7211
Simpson index	0.758	0.850	0.797	0.735	0.684	0.634	0.694	0.551	0.792	0.599	0.774	0	0.797	0.782	0.2789
Shannon index	1.894	2.206	1.891	1.659	1.372	1.125	1.649	1.239	2.032	1.470	1.803	0	1.848	1.978	0.5682

Dominance − D = Σ(n_i_/n)^2^, where n_i_ is the number of individuals of taxon i, which ranges from 0 to 1 (only one taxon in the sample); Simpson index = 1 − D.

**Table 6:** Diversity index (with respect to month of isolation).

	January	February	March	April	May	June	July	August	September	October	November	December
Number of taxa	11	14	14	16	12	17	14	14	17	14	12	12
Individuals	70	99	96	96	92	136	122	145	130	170	131	121
Dominance	0.176	0.223	0.1991	0.1877	0.2224	0.2076	0.2119	0.225	0.190	0.1578	0.216	0.194
Simpson index	0.824	0.777	0.8009	0.8123	0.7776	0.7924	0.7881	0.776	0.810	0.842	0.784	0.806
Shannon index	1.977	1.925	2.021	2.127	1.863	2.006	1.944	1.853	2.076	2.124	1.873	1.913

## Data Availability

The original contributions presented in this study are included in the article/Supplementary Material. Further inquiries can be directed to the corresponding author.

## Supplementary Materials

The following supporting information can be downloaded at: https://www.mdpi.com/article/10.3390/jof11070474/s1.
